# Risk-taking responses to crash experience: Evidence from China

**DOI:** 10.1371/journal.pone.0347543

**Published:** 2026-04-21

**Authors:** Zhixiang Fang, Haoyu Xu, Yuanni Mu

**Affiliations:** 1 School of Finance, Shanghai University of Finance and Economics, Shanghai, People’s Republic of China; 2 Shengxiang School of Business, Sanda University, Shanghai, People’s Republic of China; 3 College of Public Finance and Investment, Shanghai University of Finance and Economics, Shanghai, People’s Republic of China; Takushoku University, JAPAN

## Abstract

This study examines the effect of fund managers’ crash experience on their risk-taking behaviors. Using China’s 2007–2008 and 2014–2015 A-share market crashes, as well as the COVID-19 pandemic, as exogenous shocks, we find that managers with crash experiences significantly increased their overall risk-taking. We explain these findings using the lens of risk components, asset allocation, portfolio concentration, and incentive mechanisms. We also find that incentive mechanisms encourage these managers to take on greater risks and adopt more aggressive strategies, including increasing portfolio concentration and expanding securities holdings. These findings enrich behavioral finance theories on fund managers and provide insights for regulators to design more effective incentives and manage risk in the post-crisis periods.

## 1. Introduction

Financial markets are periodically disrupted by tail events that trigger sharp asset price declines, elevated volatility, and the rapid transmission of systemic risk. These episodes can materially reshape asset prices, market functioning, and institutional behavior [1,2]. The 2008 global financial crisis and the 2015 China A-share market crash illustrate the importance of such shocks. In the latter episode, the Shanghai Composite Index declined by more than 40% within two months, generating severe market dislocation. For mutual funds, these environments are associated with large net asset value losses, concentrated investor redemptions, and deteriorating market liquidity [3]. Fund managers must therefore make investment decisions under extreme volatility and intensified performance pressure, conditions that may have persistent effects on subsequent risk-taking. China’s mutual fund industry provides a useful setting in which to study these dynamics. By early 2025, the industry had grown to RMB 32.45 trillion in assets under management across more than 12,415 products. At the same time, recent regulatory reforms have accelerated a broader transition toward higher-quality market development. This shift has changed the opportunity set facing asset managers by reducing reliance on broad market appreciation and increasing the importance of disciplined risk-taking and alpha generation. These features make China’s mutual fund sector a natural laboratory for examining how extreme market experiences affect managerial behavior.

The central question is whether and how an exogenous market crash has persistent effects on a fund manager’s risk appetite. Prior research shows that personal experiences can shape subsequent risk preferences in lasting ways [4]. In the setting of professional asset management, however, the behavioral implications of crisis experience are theoretically ambiguous because principal-agent frictions mediate them. On the one hand, exposure to extreme losses may heighten managers’ sensitivity to tail risk and induce more conservative portfolio choices aimed at protecting career concerns and human capital. On the other hand, managers who survive severe market disruptions may become more willing to take risks as a result of desensitization [5]. TSurvivorship may therefore distort beliefs about downside risk, strengthen overconfidence, and encourage more aggressive subsequent decisions. These competing mechanisms are likely to be amplified by the institutional structure of the mutual fund industry. In particular, the convex flow-performance relationship and tournament-style evaluation system can strengthen incentives for risk-taking [6]. Following large drawdowns, managers may face an asymmetric payoff structure in which the gains from recovering performance and improving rank exceed the costs of further underperformance. Under such conditions, they may choose to gamble for resurrection by increasing portfolio risk in an attempt to generate excess returns and improve relative standing [7]. Identifying which of these mechanisms dominates after a crash is therefore important for understanding the behavioral foundations of institutional risk-taking and its implications for market stability.

Against this background, we use a panel dataset of 8,073 actively managed equity funds from July 2009 to December 2024 and construct a two-way fixed-effects panel model to systematically analyze the changes in risk-taking behavior of fund managers with a market crash experience. We find that managers with crash experience significantly increase the risk in the post-crisis period. This effect is driven primarily by an increase in idiosyncratic volatility. Mechanism analysis suggests that the post-crisis increase in risk-taking operates through two main channels. First, the rise in risk-taking is accompanied by greater stock and industry concentration, indicating a shift toward more concentrated portfolio bets. Second, the effect is stronger in environments with greater fee-based incentives, implying that managerial responses to crash experience are shaped by compensation-driven motives. In addition, flow-performance sensitivity affects the form of risk adjustment: when investor flows are weakly responsive to poor performance, managers tend to increase market beta; when flows are highly responsive to strong performance, managers instead increase tracking error and idiosyncratic volatility to enhance the prospect of outperformance.

Relative to the extant literature, this study makes three main contributions. First, it extends the behavioral finance literature on the role of personal experience in shaping risk preferences by bringing this perspective into the highly institutionalized setting of the mutual fund industry. Unlike prior studies that focus primarily on individual investors, this chapter shows that even within professional organizations characterized by formal constraints and sophisticated risk-control systems, extreme market shocks can leave persistent imprints on fund managers’ subsequent decision-making. Second, the chapter develops an integrated framework that links historical shock exposure, investment choices, and economic consequences. By connecting managers’ extreme risk experiences with compensation incentives, flow-performance convexity, and career concerns, it shows how external shocks affect institutional risk-taking through endogenous incentive channels, thereby providing new empirical evidence on agency problems in emerging capital markets. Third, the chapter offers additional informational and regulatory insights for evaluating fund idiosyncratic risk. The findings suggest that investors should assess fund risk not only through conventional performance measures, but also through managers’ prior crisis experience and the incentive environments they face. More broadly, the results imply that regulators should strengthen the monitoring of idiosyncratic risk exposures during periods of market stress and better balance performance incentives with risk constraints in compensation design, thereby promoting a more robust long-term investment framework in the mutual fund industry.

## 2. Literature review and hypotheses

### 2.1. Literature review

Fund managers, as the central agents of mutual fund operations, have their risk-taking behavior shaped by both external shocks and internal incentive mechanisms. Prior research has shown that after severe market fluctuations, particularly systemic risk events such as stock market crashes, fund managers often exhibit substantial adjustments in their investment strategies and risk profiles.

On the one hand, many researchers focus on why managers reduce portfolio risk after major shocks. The principal-agent conflict is a key source of wealth effects. A primary channel is the principal-agent conflict, wherein market downturns erode the personal wealth of managers with fund ownership stakes, thereby increasing their risk aversion and inducing more conservative asset allocations [6,8,9]. Pool et al.[10] exploiting a natural experiment based on local housing market collapses, find that managers experiencing significant housing wealth losses subsequently reduce portfolio risk exposures. Additionally, career concerns offer another explanation: managers lower their portfolio’s idiosyncratic risk and tracking error to avoid dismissal. From a psychological perspective, catastrophic events can induce fear and anxiety that elevate managers’ risk aversion through emotional contagion and cognitive bias. Bernile [11] documents that even fund managers who haven’t suffered actual financial losses will exhibit significantly more conservative behavior following traumatic events. Similarly, Cohn et al. [12] also show that fear induced by hypothetical scenarios can lower investors’ willingness to bear risk. The limited-attention theory further complements this view: major market disruptions or personal life shocks may distract managers from investments, weakening their ability to process information and active management capabilities. Thus, they may shift toward lower-risk strategies, such as indexing or passive investment approaches [13].

On the other hand, a growing body of research indicates that some fund managers may increase their risk exposure following a market shock. From the perspective of incentive structures, extreme market fluctuations often amplify the motivation for performance recovery. In such cases, managers may adopt more aggressive risk-taking strategies, such as increasing idiosyncratic risk or portfolio concentration, in an attempt to capture excess returns during a potential market rebound [14,15]. This “revenge-motivated” behavior is particularly evident in an environment with high performance-flow sensitivity, where stronger investor reactions to top-tier performance incentivize managers to pursue higher risks to preserve or expand assets under management and associated fee income. In addition, psychological mechanisms may also drive risk-seeking behavior. After experiencing a stock market crash or other major events, some managers may perceive the likelihood of another extreme event as low, leading them to view the post-crisis market as offering undervalued opportunities. Consequently, they may be more willing to take on risks to seize what they see as undervalued opportunities during the market recovery period. Moreover, although the geographical proximity studies by Dessaint and Matray [16] and Alok et al. [17] primarily highlight disaster-induced avoidance behaviors, they also reveal that under information asymmetry and the availability heuristic, managers may have overly optimistic expectations when assessing opportunities and risks, thus increasing their risk-taking.

Overall, whether driven by active responses to performance-based incentives or agency-motivated behavior triggered by external wealth shocks, fund managers tend to adjust their portfolio risk levels after experiencing systemic market turbulence such as a stock market crash. These adjustments do not necessarily reflect a shift toward risk aversion. Instead, they may demonstrate a higher inclination to take risks, especially when fund managers aim to repair their performance or compensate for wealth losses through a “high-risk, high-reward” strategy.

### 2.2. Hypotheses

Current studies indicate that after experiencing systemic market shocks, such as a market crash, fund managers often adjust their investment strategies in pursuit of a rapid performance rebound. Bernile finds that the disaster experience itself can alter managers’ risk attitudes through an emotional mechanism, leading them to be more aggressive in asset allocation [11]. The cross-country empirical evidence from Dannhauser and Spilker [18] further confirms that when compensation incentives are strong, fund managers increase their holdings of high-β assets after market downturns to boost performance rankings and personal compensation through excess returns. Also, in the context of agency conflicts, a compensation-driven motive plays a key role. Guercio and Reuter [9] demonstrate that when fund managers’ wealth is closely tied to the fund they manage, losses incurred during a crash may incentivize them to increase portfolio risk in an attempt to recoup asset shrinkage. Luo et al. [19] find that after a crisis, some fund managers are more likely to select stocks and make tactical adjustments actively. Based on this, we propose the following hypothesis:

*Hypothesis 1: Fund managers who have experienced a market crash will increase their overall risk-taking in subsequent investments.*

Fund risk is fundamentally shaped by both asset allocation and portfolio structure. Holding concentration serves as a key metric for evaluating active management and risk profiles. Specifically, higher portfolio concentration indicates a greater active deviation from benchmark indices [20]. While diversification mitigates idiosyncratic risk during stable periods, extreme market shocks often prompt managers to restructure portfolios and intensify active management. Elevated industry concentration reflects managerial confidence and a strategic attempt to exploit private information [15]. Following extreme risk events, managers seek differentiated performance by concentrating capital in familiar industries or highly elastic assets [21]. This deliberate narrowing of the investment scope abandons traditional diversification. Consequently, it significantly increases idiosyncratic risk and return volatility. Furthermore, this shift toward high concentration acts as more than just an informational strategy. Under intense ranking evaluations and redemption pressures, it serves as a covert risk-taking mechanism driven by agency conflicts, enabling managers to gamble for performance reversals. Based on this, we propose the following concentration mechanism hypothesis:

*Hypothesis 2: The increase in risk-taking is affected by higher investment concentration in stocks or industries.*

Fund managers’ risk-taking behavior is primarily driven by incentive mechanisms, particularly compensation structures, which significantly impact their investment decisions. According to agency theory, mutual fund companies commonly link compensation, such as management fees and performance-based bonuses, to investment outcomes, thereby encouraging managers to assume higher risks in pursuit of excess returns [22]. A substantial body of research has shown a significant connection between fund size, fee structures, and risk-taking. Kempf et al. [23] find that managers with lower interim performance rankings tend to significantly increase portfolio risk in the latter half of the evaluation period, reflecting the impact of non-linear compensation structures. Similarly, Farnsworth and Taylor [24] point out that fund managers’ bonuses are closely tied to management fees, and the income expectation from fund size expansion can strengthen risk-taking preferences. Dannhauser and Spilker [18] provided cross-country empirical evidence that, under high-intensity incentives, managers tend to allocate more capital to high-β assets following a market downturn in order to improve their rankings. Moreover, Guercio and Reuter [9] find that strong crisis performance can initiate a “compensation-risk” positive feedback loop. Managers who perform well during market crashes often receive greater investor recognition and attract substantial capital inflows. And the resulting increase in management fees further motivates these managers to take on greater risks in subsequent periods.

Therefore, if fund managers demonstrate strong resilience during major market shocks, they are more likely to gain greater recognition from investors and fund companies, resulting in increased capital inflows and higher management fee income, which in turn reinforces their incentives to take on additional risk. Based on this, we propose the following hypothesis:

*Hypothesis 3: Extreme risk experiences influence fund managers’ risk-taking behavior through incentive mechanisms.*

## 3. Data and methodology

### 3.1. Data

Our sample consists of actively managed Chinese domestic open-end equity and hybrid funds. The data are primarily obtained from the China Stock Market & Accounting Research Database (CSMAR) and the Wind Database (WIND), from July 2009 to December 2024. We obtain fund net-of-fee accumulative returns, adjusted for historical fund payouts and splits, from the CSMAR database, and fund characteristics such as expense ratios and the total net assets (TNA) of funds and fund families from the WIND database. We use fund tickers to merge the CSMAR and WIND databases. We exclude QDII, ETF, structured, and umbrella funds, and remove samples where the top 20 holdings account for less than 20% of the net assets. After these steps, we obtained 4,846 valid samples. To avoid the impact of extreme values, we applied a 1% winsorization to the data.

### 3.2. Variables

#### 3.2.1. Crash experience.

If a fund manager has experienced any of the extreme risk events during their career, including the extreme risks in 2007−2008, 2014−2015, or the COVID-19 pandemic, they are deemed to have extreme risk experience. For funds managed by a team, the maximum exposure is adopted. The specific calculation method is as follows:


$$\begin{array}{c} {\text{Crash}}_{\text{i},\text{t}}=\left\{ \begin{array}{c} @l\text{1},max\left(\overset{{\text{N}}_{\text{i},\text{t}}}{\underset{\text{n}=\text{1}}{\left\{{\text{Expdate}}_{\text{n}}\right\}}}\right)\geq \text{3}\text{0}\text{0}\;\\ \text{1},max\left(\overset{{\text{N}}_{\text{i},\text{t}}}{\underset{\text{n}=\text{2}}{\left\{{\text{Expdate}}_{\text{n}}\right\}}}\right)\geq \text{1}\text{8}\text{0}\\ \text{1},max\left(\overset{{\text{N}}_{\text{i},\text{t}}}{\underset{\text{n}=\text{3}}{\left\{{\text{Expdate}}_{\text{n}}\right\}}}\right)\geq \text{4}\text{0}\text{0}\;\\ \text{0},\;otherwise \end{array}\right.\; \end{array}$$ (1)

Where ${\text{Crash}}_{\text{i},\text{t}}$= 1 indicates that fund i at time t is managed by at least one fund manager who has experienced at least one extreme risk event, and $N_{i,t}$ is the number of people co-managing the fund at the same time. n = 1,2,3 correspond to the three types of extreme risk events respectively. A fund manager is considered to have experience in the 2007−2008 extreme risk if they were in office for more than 300 days during the period from October 16, 2007 to October 28, 2008 (379 days). Secondly, a fund manager is regarded as having experience in the 2014−2015 extreme risk if they were in office for more than 180 days during the period from June 12, 2015 to January 28, 2016 (231 days). Thirdly, if they were in office for 400 days or more during the period from December 9, 2019 to June 30, 2021 (570 days), they are considered to have experience in the COVID-19 pandemic.

#### 3.2.2. Fund risk-taking.

Following Pool et al. [10], we measure fund risk using the semi-annual standard deviation of weekly returns. Fund risk is expressed as:


$$\begin{array}{c} \text{Ris}k_{i,t}=\sum_{i=\text{1}}^{N}\overset{f}{\underset{i,t}{R}} \end{array}$$ (2)

#### 3.2.3. Control variables.

Following previous studies [7,14,23,25,26], we include a comprehensive set of fund- and manager-level control variables in our regression models to account for other factors influencing fund risk. Table 1 provides detailed definitions for all variables.

**Table 1:** Main control variables.

Category	Variable	Definition
Fund Manager	Tenure	For funds managed by a team, we use the longest tenure of the team as the fund manager’s age. Tenure indicates the longest tenure among team members. Certificate indicates whether at least one manager in the team has passed one of the three exams. PhD indicates whether at least one manager holds a Ph.D. degree.
	Gender	
	Certificate	
	PhD	
Fund	Ret	The fund’s raw return over six months.
	FundSize	Natural logarithm of fund size
	Flow	$\text{Flo}w_{i,t}=\frac{TNA_{i,t}}{TNA_{i,t-\text{1}}}-\left(\text{1}+R_{i,t}\right)$
	FundAge	Number of years since the fund’s inception.
	Turnover	Total fund purchases and sales are divided by the minimum TNA.
	FamilySize	Natural logarithm of the fund family’s total assets under management.

### 3.3. Research model

To test whether fund managers exposed to a market crash increase the risk-taking of their managed funds, we build a two-way fixed-effects panel data model as follows:


$$\begin{array}{c} {Risk}_{i,t}=\beta_{\text{0}}+\beta_{\text{1}}{Crash}_{i,t-\text{1}}+\beta_{\text{2}}{Control}_{i,t-\text{1}}+Q_{t}+\lambda_{t}+\varepsilon_{i,t} \end{array}$$ (3)

Where ${Risk}_{i,t}$ is the fund *i*’s risk level in period *t*, measured by the standard deviation of weekly fund re*t*urns over a semi-year period. ${Crash}_{i,t}$ equals 1 if fund manager *i* experienced the 2007–2008 crash for over 300 days, and 0 otherwise. ${Control}_{i,t-\text{1}}$ include fund and manager-level controls. $Q_{t}$ Year fixed effects. $\lambda_{t}$ Industry fixed effects. $\;\varepsilon_{i,t}$ is error term. To mitigate endogeneity, we use lagged control variables, including fund size, volatility, age, family size, dividends, manager experience, and highest degree. In our regression analysis, we include both manager and semi-year fixed effects. Standard errors are clustered at the fund level.

### 3.4. Descriptive statistics

Our final sample comprises 8,073 public mutual funds from July 2009 to December 2024, yielding 69,015 fund-half-year observations. As reported in Table 2, the average risk level for funds managed by individuals with prior crash experience (Crash = 1) is 0.097, which is significantly higher than the 0.090 observed for the control group (Crash = 0). This preliminary finding indicates that exposure to extreme market shocks does not induce conservative investment behavior; rather, it elevates managerial risk-taking. Regarding other characteristics, the Crash = 1 group exhibits significantly greater average fund age and asset size, suggesting that managers with crisis experience are typically entrusted with larger, more established funds. Furthermore, these managers possess significantly longer industry tenures and a higher proportion of advanced degrees.

**Table 2:** Summary statistics.

	All Sample		Crash = 0		Crash = 1			
	(N = 69015)		(N = 38020)		(N = 30995)			
	Mean	Median	Mean	Median	Mean	Median	Mean diff.	t-statistics
Risk	0.093	0.087	0.090	0.085	0.097	0.090	0.007^***^	(15.027)
Ret	0.011	0.005	−0.015	−0.013	0.042	0.029	0.044^***^	(50.240)
Flow	0.719	−0.073	0.650	−0.082	0.796	−0.063	0.100^***^	(2.959)
FundAge	3.532	3.664	3.212	3.258	3.924	4.060	0.569^***^	(98.428)
FundSize	1.094	1.201	0.902	0.993	1.329	1.430	0.559^***^	(40.992)
FamilySize	7.290	7.374	7.312	7.410	7.263	7.343	0.370^***^	(25.515)
Dividend	0.008	0.000	0.005	0.000	0.011	0.000	0.006^***^	(21.188)
Tenure	2.969	3.091	2.588	2.708	3.433	3.611	0.788^***^	(142.861)
Gender	0.217	0.000	0.213	0.000	0.221	0.000	0.008^**^	(2.459)
Certificate	0.053	0.000	0.051	0.000	0.055	0.000	0.004^**^	(2.120)
Degree	0.134	0.000	0.123	0.000	0.148	0.000	0.025^***^	(9.543)

## 4. Empirical results

### 4.1. Baseline results

Table 3 presents the estimation results of Equation (3), examining how market crash experience affects fund managers’ risk-taking. Column (1) presents the results, controlling only for time and individual effects. Column (2) adds fund-level controls. Column (3) further includes fund manager-level controls. Column (4) further includes contemporaneous fund performance to control for potential performance reversals and lagged fund risk to account for potential autocorrelation.

**Table 3:** Crash experience and risk-taking of mutual fund managers.

	(1)	(2)	(3)	(4)
	Risk_t_	Risk_t_	Risk_t_	Risk_t_
Crash_t-1_	0.0069^***^	0.0024^***^	0.0028^***^	0.0015^***^
	(12.39)	(3.12)	(3.54)	(2.89)
Riskt_t-1_				0.4413^***^
				(69.75)
Ret_t-1_		0.0451^***^	0.0448^***^	0.0190^***^
		(17.67)	(17.62)	(8.65)
Flow_t-1_		−0.0001^***^	−0.0001^***^	−0.0000
		(−3.19)	(−2.91)	(−1.12)
FundAge_t-1_		0.0018^***^	0.0020^***^	0.0009^***^
		(4.62)	(4.91)	(3.48)
FundSize_t-1_		−0.0003^*^	−0.0003^*^	−0.0001
		(−1.70)	(−1.75)	(−1.06)
FamilySize_t-1_		−0.0020^***^	−0.0020^***^	−0.0016^***^
		(−3.73)	(−3.60)	(−4.23)
Dividend_t-1_		−0.0015	−0.0015	0.0060
		(−0.35)	(−0.35)	(1.51)
Tenure_t-1_			−0.0005^*^	−0.0002
			(−1.72)	(−0.73)
Gender_t-1_			−0.0156^***^	−0.0094^***^
			(−9.11)	(−8.21)
Certificate_t-1_			0.0011	−0.0009
			(0.39)	(−0.46)
Degree_t-1_			−0.0006	−0.0004
			(−0.31)	(−0.35)
Individual FE	Yes	Yes	Yes	Yes
Time FE	Yes	Yes	Yes	Yes
_cons	0.0869^***^	0.0999^***^	0.1034^***^	0.0624^***^
	(282.35)	(24.00)	(24.61)	(20.61)
N	102593	62435	61925	61898
R^2^	0.6457	0.6893	0.6910	0.7537

*t* statistics in parentheses.

* *p* < 0.1, ** *p* < 0.05, *** *p* < 0.01.

Across all specifications, the coefficient on Crash_t-1_ is positive and statistically significant at the 1% level. Economically, the Column (4) estimates indicate that managers with crash experience increase their total portfolio risk by an average of 0.15 percentage points relative to their unexposed peers. These results strongly support our core hypothesis: surviving an extreme market shock does not heighten risk aversion, but rather induces greater risk-taking. This yields novel evidence from the Chinese market that Malmendier and Nagel’s theory of experience-driven risk preferences extends robustly to professional institutional investors [4].

The control variables align with existing literature. The coefficient on lagged fund return (Ret_t-1_) is significantly positive, suggesting that recent winners tend to take on more risk [27]. Conversely, fund size (FundSize_t-1_) and family size (FamilySize_t-1_) load negatively, reflecting the risk-constraining effects of liquidity limits and stringent internal controls at larger institutions [25]. Finally, consistent with Niessen-Ruenzi and Ruenzi [28], the negative coefficient on Gender_t-1_ indicates that female managers exhibit comparatively greater risk aversion.

### 4.2. Robustness test

In the empirical analysis, we controlled for potential earnings reversals and autocorrelation by including contemporaneous fund performance and lagged fund risk. We also employed a two-way fixed-effects panel regression model. Additionally, we conducted further robustness tests.

#### 4.2.1. Instrumental variable test.

In this study, we investigate the effect of extreme risk experiences on managers’ risk-taking choices. To address potential endogeneity concerns, we estimate a two-stage least squares (2SLS) model using an indicator of whether a manager’s initial tenure year predates the crisis as an instrumental variable (IV_crash_) [29]. This instrument is highly relevant, as entry timing dictates exposure probability. It also satisfies the exclusion restriction, since exogenous factors like birth year primarily determine career entry timing, which does not directly affect current risk preferences after controlling for tenure.

Table 4 reports the results. The first-stage estimates in Column (1) confirm the instrument’s validity: IV_crash_ is highly significant, and the Kleibergen-Paap statistics firmly rule out underidentification and weak instrument concerns. In the second stage (Column 2), the coefficient on extreme risk experience remains significantly positive. Therefore, after correcting for endogeneity bias, our core conclusion remains robust: managers experiencing a market crash significantly increase managerial risk-taking.

**Table 4:** Instrumental variable test.

	(1)	(2)
	Crash_t_	Risk_t_
IV_crash_	0.8865^***^	
	(0.0056)	
Crash_t-1_		0.0023^**^
Controls_t-1_	Yes	Yes
Individual FE	Yes	Yes
Time FE		
*N*	60235	60235
r2	0.8544	0.0075
Kleibergen-Paap rkLM	2473.87^***^	
	(0.0000)	
Kleibergen-Paap rk Wald F	25188.89	

Standard errors in parentheses.

* *p* < 0.10, ** *p* < 0.05, *** *p* < 0.01.

#### 4.2.2. PSM.

To address potential sample selection bias, we further conduct a robustness check using the Propensity Score Matching (PSM) method. Given the exogeneity of the 2007–2008 crash, we partition the samples into a treatment group (managers appointed pre-crash) and a control group ((managers appointed post-crash). Then, based on the observable characteristics of fund managers without crash experience, we construct a matching model and pair each treated manager with up to four control managers sharing the most similar characteristics. Specifically, Model (1) calculates propensity scores using fund-level controls, including returns, flows, size, and dividends. Model (2) uses manager-level controls, including tenure, gender, certification, and education. Model (3) incorporates both fund- and manager-level characteristics. This matching procedure allows us to better isolate the causal effect of crash exposure on subsequent managerial risk-taking.

As reported in Table 5, the empirical findings remain consistent with the baseline results, regardless of the matching specification used. The crash experience induces managers to adjust their investment strategies, increasing risk exposure and thus leading to a rise in fund risk levels. This confirms the robustness of the baseline regression results.

**Table 5:** PSM.

	Risk_t_			
		Model(1)	Model(2)	Model(3)
N = 1	Crash_t-1_	0.0025^**^	0.0027^***^	0.0023^**^
		(2.49)	(3.36)	(2.18)
	Controls_t-1_	Yes	Yes	Yes
	Individual FE	Yes	Yes	Yes
	Time FE	Yes	Yes	Yes
	_cons	0.1050^***^	0.1047^***^	0.0989^***^
		(17.70)	(23.64)	(15.44)
	*N*	27037	58981	21399
	*R* ^2^	0.693	0.692	0.703
	Crash_t-1_	0.0024^***^	0.0027^***^	0.0024^**^
N = 2		(2.73)	(3.36)	(2.51)
	Controls_t-1_	Yes	Yes	Yes
	Individual FE	Yes	Yes	Yes
	Time FE	Yes	Yes	Yes
	_cons	0.1092^***^	0.1047^***^	0.1027^***^
		(21.64)	(23.64)	(18.86)
	*N*	39394	58986	31255
	*R* ^2^	0.691	0.692	0.696
	Crash_t-1_	0.0025^***^	0.0027^***^	0.0027^***^
N = 3		(2.96)	(3.36)	(3.04)
	Controls_t-1_	Yes	Yes	Yes
	Individual FE	Yes	Yes	Yes
	Time FE	Yes	Yes	Yes
	_cons	0.1084^***^	0.1046^***^	0.1024^***^
		(22.32)	(23.62)	(20.22)
	*N*	46137	58994	37496
	*R* ^2^	0.693	0.692	0.692
	Crash_t-1_	0.0026^***^	0.0027^***^	0.0028^***^
N = 4		(3.13)	(3.37)	(3.21)
	Controls_t-1_	Yes	Yes	Yes
	Individual FE	Yes	Yes	Yes
	Time FE	Yes	Yes	Yes
	_cons	0.1060^***^	0.1046^***^	0.1051^***^
		(22.69)	(23.63)	(21.71)
	*N*	50370	59005	41939
	*R* ^2^	0.693	0.692	0.691

Standard errors in parentheses.

* *p* < 0.1, ** *p* < 0.05, *** *p* < 0.01.

#### 4.2.3. Alternative crash experience measurement.

To assess the robustness of our baseline regression estimates, we construct a continuous measure of market crash exposure based on the total number of days a manager was exposed to the three market crashes. Specifically, we estimate the specifications using the mean values of fund-level manager data (columns (1)-(4)) and the median values (columns (5)-(8)) of this exposure metric at the fund level. As shown in Table 6, the estimation results from this continuous measurement approach are consistent with the baseline findings.

**Table 6:** Alternative crash experience measurement.

	Risk_t_	
Aggregate function	Mean	Median
	(1)	(2)
Crash_t-1_	7.61e-06^*^	7.22e-06^*^
	(1.74)	(1.66)
Controls_t-1_	Yes	Yes
Individual FE	Yes	Yes
Time FE	Yes	Yes
N	61925	61925
R^2^	0.6908	0.6908

Standard errors in parentheses.

* *p* < 0.1, ** *p* < 0.05, *** *p* < 0.01.

## 5. How do fund managers increase risk?

### 5.1. Risk components

To further examine whether extreme risk exposure alters the structure of managerial risk-taking, we decompose total risk into three dimensions: systematic risk (Beta_t_), idiosyncratic volatility (Idvol_t_), and tracking error (Tracking Error_t_). First, based on the Capital Asset Pricing Model (CAPM), we regress weekly excess fund returns on the market risk premium. The coefficient on the market factor serves as the measure for systematic risk, and the volatility of the regression residuals measures idiosyncratic risk. Additionally, because domestic mutual funds operate under strict relative performance evaluations, we construct the tracking error metric using the standard deviation of the fund’s weekly return differentials relative to the Shanghai Composite Index to capture the degree of deviation from the performance benchmark.

The results are reported in Table 7. Similar to the baseline regressions, we progressively add controls from a model with only time and individual fixed effects in Column (1) to a fully specified model in Column (3). The results indicate that across all specifications, the coefficients of Crash_t-1_ on Beta_t_ and Idvol_t_ are positive and significant at the 1% level, consistent with the increase in total risk presented in Table 3. These findings suggest that managers exposed to extreme risk shocks subsequently adopt a dual-dimension risk strategy: they elevate market risk exposure to capture the systematic risk premium, while simultaneously exhibiting a stronger propensity for active stock selection to pursue excess returns [30].

**Table 7:** Crash experience and risk-taking: Risk components.

	(1)	(2)	(3)
	Beta_t_		
Crash_t-1_	0.0347^***^	0.0428^***^	0.0347^***^
	(7.54)	(7.29)	(5.88)
Fund Controls_t-1_	No	Yes	Yes
Manager Controls_t-1_	No	No	Yes
Individual FE	Yes	Yes	Yes
Time FE	Yes	Yes	Yes
_cons	0.6031^***^	0.8967^***^	0.9031^***^
	(248.58)	(32.46)	(33.02)
N	88928	62442	61932
R^2^	0.5114	0.5916	0.5937
	Idvol_t_		
Crash_t-1_	0.0026^***^	0.0053^***^	0.0046^***^
	(4.46)	(6.69)	(5.91)
Fund Controls_t-1_	No	Yes	Yes
Manager Controls_t-1_	No	No	Yes
Individual FE	Yes	Yes	Yes
Time FE	Yes	Yes	Yes
_cons	0.0740^***^	0.1117^***^	0.1121^***^
	(244.36)	(27.43)	(27.56)
N	88822	62442	61932
R^2^	0.6118	0.6909	0.6930
	Tracking Error_t_		
Crash	−0.0009	0.0166	0.0153
	(−0.11)	(1.53)	(1.37)
Fund Controls_t-1_	No	Yes	Yes
Manager Controls_t-1_	No	No	Yes
Individual FE	Yes	Yes	Yes
Time FE	Yes	Yes	Yes
_cons	1.7237^***^	1.8841^***^	1.8798^***^
	(378.66)	(28.50)	(28.22)
N	79023	62219	61710
R^2^	0.5567	0.5803	0.5807

*t* statistics in parentheses.

* *p* < 0.1, ** *p* < 0.05, *** *p* < 0.01.

As for tracking error, the coefficient on Crash_t-1_ is not statistically significant, suggesting that while crash-experienced fund managers may increase idiosyncratic risk, they do not substantially deviate from market benchmarks. This contrasts interestingly with Chevalier and Ellison [14], who found that managers under career-related pressure reduce idiosyncratic risk and exhibit more herding behavior. However, we find that in China, fund managers with crash experience employ more active strategies by increasing idiosyncratic risk to enhance potential returns, rather than shifting toward more conservative behavior.

### 5.2. Shifts in portfolio composition

Following the evidence that crash exposure alters managerial risk-taking, we now examine the specific shifts in portfolio structure that drive this effect. Theoretically, fund managers can increase fund risk through various means, such as upping the number of stocks held, adjusting cash allocations, lowering the weight of top-held securities, or boosting industry diversification. However, these different mechanisms influence risk-taking in distinct ways. To comprehensively examine their actual effects, we use data from the Wind Database to calculate each fund’s number of stocks held, portfolio concentration, industry concentration, and allocation weights for securities, cash, and bonds. Portfolio concentration is measured by the Herfindahl index, while industry concentration follows the approach of Kacperczyk et al. [15].

Table 8 shows that fund managers with extreme risk shocks exhibit significant structural adjustments in their investment strategies. In Column (1), the coefficient is positive but statistically insignificant. In Column (2), the coefficient on the industry concentration variable ($\overset{\text{Industry}}{\underset{\text{t}}{\text{HHI}}}$) is significantly positive at the 1% level, suggesting that managers tend to concentrate their investments in fewer industries after experiencing extreme risk events. This industry-level concentration further increases the portfolio’s sensitivity to sector-specific fluctuations, thereby amplifying idiosyncratic risk even when the overall exposure to systematic risk remains unchanged. Columns (3) to (5) examine the reallocation across major asset classes, including equities, cash, and bonds. The results show a significant positive relationship between Crash and the equity allocation ratio (Equityₜ), indicating that managers with extreme risk experience are more likely to increase their exposure to equity assets, thereby raising portfolio volatility and risk exposure. Moreover, Crashₜ_-1_ is significantly negatively associated with the bond allocation ratio (Bondₜ), further proving a shift toward riskier assets. This shift—increasing equities while decreasing bonds—clearly shows that extreme risk experience does not lead managers toward conservative defensive assets like bonds to hedge against uncertainty. Instead, they tilt their portfolios toward high-volatility, high-elasticity assets, substantially expanding their risk exposure. This portfolio rebalancing toward riskier assets provides a direct mechanism for the significant increase in systematic risk (Betaₜ) observed earlier.

**Table 8:** Shifts in portfolio composition.

	(1)	(2)	(3)	(4)	(5)
	$\overset{𝐇𝐨𝐥𝐝𝐢𝐧𝐠}{\underset{𝐭}{𝐇𝐇𝐈}}$	$\overset{𝐈𝐧𝐝𝐮𝐬𝐭𝐫𝐲}{\underset{𝐭}{𝐇𝐇𝐈}}$	Equity_t_	Cash_t_	Bond_t_
Crash_t-1_	0.0032	0.0010^***^	1.6890^***^	−0.0746	−1.6971^***^
	(0.94)	(3.79)	(4.09)	(−0.48)	(−2.90)
Controls_t-1_	Yes	Yes	Yes	Yes	Yes
Individual FE	Yes	Yes	Yes	Yes	Yes
Time FE	Yes	Yes	Yes	Yes	Yes
_cons	0.5917^***^	0.0310^***^	68.1839^***^	12.4975^***^	28.6193^***^
	(30.70)	(20.95)	(40.35)	(13.84)	(10.06)
N	61926	51835	61947	61947	37034
R^2^	0.4073	0.6339	0.7519	0.3619	0.7823

*t* statistics in parentheses.

* *p* < 0.1, ** *p* < 0.05, *** *p* < 0.01.

### 5.3. Incentive mechanism

To explore the underlying mechanisms, we test whether compensation incentives drive fund managers to take on more risk after the crash. Managers who survive severe market shocks may signal superior risk-management capabilities to the market. This signal attracts capital inflows, which significantly increases their assets under management (AUM) and compensation levels [19,31,32]. In the mutual fund industry, convex incentive structures and rank-based tournament competition further encourage these managers to take higher risks to pursue excess returns [6,33]. Since individual compensation data for Chinese fund managers is unavailable, we use fund management fees as a proxy for incentive levels. In China, compensation is closely linked to AUM, and management fees determine the funding available for salaries and bonuses. To test the path from “crash experience → increased incentives → higher risk-taking,” we construct a mediation effect model. Specifically, we first estimate the total effect of crash exposure on current risk-taking (Model 4). Second, we examine the impact of crash exposure on the mediator, management fees (Model 5). Finally, we include both the core explanatory variable and the lagged mediator (Fee_t-1_) in the risk-taking regression (Model 6) to identify the mediating role of compensation incentives. The equations are as follows:


$$\begin{array}{c} Risk_{i,t}=\beta_{\text{0}}+\beta_{\text{1}}\;Crash_{i,t-\text{1}}+\beta_{\text{2}}\;X_{i,t-\text{1}}+\mu_{i}+\nu_{t}+\epsilon_{i,t} \end{array}$$ (4)

$$\begin{array}{c} {Fee}_{i,t}=\alpha_{\text{0}}+\alpha_{\text{1}}\;Crash_{i,t-\text{1}}+\alpha_{\text{2}}\;X_{i,t-\text{1}}+\mu_{i}+\nu_{t}+\varepsilon_{i,t} \end{array}$$ (5)

$$\begin{array}{c} Risk_{i,t}=\gamma_{\text{0}}+\gamma_{\text{1}}\;Crash_{i,t-\text{1}}+\gamma_{\text{2}}\;{Fee}_{i,t-\text{1}}+\gamma_{\text{3}}\;X_{i,t-\text{1}}+\mu_{i}+\nu_{t}+\epsilon_{i,t} \end{array}$$ (6)

The regression results are shown in Table 9. First, Model (1) shows that extreme risk exposure significantly increases fund risk-taking. Second, Model (2) shows that the coefficient of Crash_t-1_ on Fee_t-1_ is 0.1010 and significantly positive at the 1% level. This indicates that managers exposed to extreme risk shocks earn higher management fees, supporting the theoretical expectation that crash exposure strengthens compensation incentives. Finally, in Model (3), the coefficient on Crash_t-1_ drops from 0.0028 to 0.0009, while the coefficient on Fee_t-1_ is 0.0007, significant at the 1% level. This finding suggests that the impact of crash exposure on subsequent risk-taking is not driven by a direct shift in managerial risk preferences. Instead, it operates through the stable income expectations generated by continued fee revenue after a crisis, which provides managers with a risk-taking buffer.

**Table 9:** Incentive mechanism.

	(1)	(2)	(3)
	Risk_t_	Fee_t_	Risk_t_
Crash_t-1_	0.0028^***^	0.1010^***^	0.0009
	(3.54)	(3.63)	(0.99)
Fee_t-1_			0.0007^***^
			(3.47)
Controls_t-1_	Yes	Yes	Yes
Individual FE	Yes	Yes	Yes
Time FE	Yes	Yes	Yes
_cons	0.1034^***^	−0.5262^***^	0.1052^***^
	(24.61)	(−2.88)	(22.63)
N	61925	52170	48787
R^2^	0.6910	0.7275	0.6957

t statistics in parentheses.

* *p < 0.1,* ** *p < 0.05,* *** *p < 0.01.*

### 5.4. Heteroecity analysis: Performance-flow sensitivity

To test whether the impact of crash exposure on managerial risk-taking varies across different incentive structures, we examine the heterogeneity in Performance-Flow Sensitivity (PFS). Specifically, we construct an interaction term between PFS and Crash_t-1_ to test for differential risk-taking responses following severe market shocks.

Following standard literature, we model the asymmetric response of fund flows to past performance using a piecewise linear regression framework [10,34]. First, we estimate the following model for each fund to measure PFS:


$$\begin{array}{c} Flow_{j,t}\;=\;f\left(Perf_{j,t-\text{1}}\right)\;+\;{\text{Controls}}_{j,t-\text{1}}\;+\;\varepsilon_{j,t} \end{array}$$ (7)

Where $Flow_{j,t}\;$ denotes the net flow ratio of fund j in month t. $Perf_{j,t-\text{1}}$ is measured by the fund j’s alpha in month t-1, estimated from a CAPM regression using the fund’s monthly returns over the past 36 months. Control variables include lagged flow, fund size, age, and fees. In this piecewise linear model, we partition $Perf_{j,t-\text{1}}$ into three segments: the lowest performance quintile (${\text{LowPerf}}_{j,t-\text{1}}=\text{min}\left(Perf_{j,t-\text{1}},\text{0}.\text{2}\right)$), the middle three performance quintiles (${\text{MidPerf}}_{j,t-\text{1}}=\text{min}\left(\text{max}\left(Perf_{j,t-\text{1}}-\text{0}.\text{2},\text{0}\right),\text{0}.\text{6}\right)$), and the highest performance quintile (${\text{HighPerf}}_{j,t-\text{1}}=\text{max}\left(Perf_{j,t-\text{1}}-\text{0}.\text{8},\text{0}\right)$). We then use the coefficient on the bottom and top performance segments to construct the “Low performance-flow sensitivity” (LPFS) and “High performance-flow convexity” (HPFC) indicators, respectively.

Table 10 reports the estimation results. In the low performance-flow sensitivity subsample (columns (1)-(4)), the coefficient on the interaction term LPFS_t-1_ × Crash_t-1_ is statistically insignificant across all risk metrics. This means that weak flow penalties for poor performance do not further stimulate risk-taking among crash-exposed managers. In contrast, within the high-performance-flow convexity subsample (columns (5)-(8)), HPFC_t-1_ × Crash_t-1_ is significantly positive for both total risk (Risk_t_) (0.0100 at the 5% level) and idiosyncratic volatility (Idvol_t_) (0.0057 at the 10% level). These results show that highly convex incentives amplify risk-taking among crash-exposed managers. Driven by the massive capital inflows awarded to top performers, these managers increase idiosyncratic risk to secure top rankings. Overall, this evidence further corroborates our earlier findings on the compensation incentive channel.

**Table 10:** Performance-flow sensitivity.

	(1)	(2)	(3)	(4)
	Risk_t_	Beta_t_	Idvol_t_	Tracking error_t_
Crash_t-1_	0.0023	0.0099	0.0015	0.0331
	(1.51)	(1.32)	(1.26)	(1.32)
LPFS_t-1_	−0.0010	−0.0074	−0.0025^**^	−0.0323
	(−0.69)	(−0.92)	(−2.04)	(−1.23)
LPFS_t-1_ × Crash_t-1_	0.0005	−0.0029	−0.0010	0.0135
	(0.27)	(−0.28)	(−0.61)	(0.40)
Controls_t-1_	Yes	Yes	Yes	Yes
Individual FE	Yes	Yes	Yes	Yes
Time FE	Yes	Yes	Yes	Yes
_cons	0.0866^***^	0.5118^***^	0.0584^***^	1.5132^***^
	(5.67)	(7.39)	(5.21)	(6.19)
N	12735	12739	12739	12717
R^2^	0.6580	0.6949	0.7311	0.5812
	**(5)**	**(6)**	**(7)**	**(8)**
	**Risk**_**t**_	**Beta**_**t**_	**Idvol**_**t**_	**Tracking error**_**t**_
Crash_t-1_	0.0021	0.0093	0.0011	0.0339
	(1.48)	(1.33)	(1.38)	(1.43)
HPFC_t-1_	−0.0061	0.0011	−0.0053^**^	−0.0027
	(−1.61)	(0.05)	(−2.01)	(−0.04)
HPFC_t-1_ × Crash_t-1_	0.0100^**^	−0.0178	0.0057^*^	0.0590
	(2.22)	(−0.60)	(1.77)	(0.79)
Controls_t-1_	Yes	Yes	Yes	Yes
Individual FE	Yes	Yes	Yes	Yes
Time FE	Yes	Yes	Yes	Yes
_cons	0.0876^***^	0.5148^***^	0.0337^***^	1.5407^***^
	(5.74)	(7.42)	(4.28)	(6.30)
N	12735	12739	12739	12717
R^2^	0.6582	0.6949	0.7853	0.5812

t statistics in parentheses.

* *p < 0.1,* ** *p < 0.05,* *** *p < 0.01.*

### 5.5. Heteroecity analysis: Manager characteristics

Our baseline results have indicated that crash experiences generally increase fund managers’ risk-taking. However, behavioral finance literature documents that individual characteristics also influence financial decision-making [4,35]. For example, educational background serves as a proxy for information-processing capacity [14], gender is associated with differences in risk tolerance [36], and age relates to career concerns [37]. To examine this cross-sectional heterogeneity, we test whether managers’ education, gender, industry tenure, and fund size condition their risk-taking responses to market crashes. Specifically, we introduce interaction terms into the baseline specification and estimate the following model:


$${Risk}_{i,t}=\beta_{\text{0}}+\beta_{\text{1}}{Crash}_{i,t-\text{1}}\times Characteristic_{i,t-\text{1}}+\beta_{\text{2}}{Crash}_{i,t-\text{1}}$$

$$+\beta_{\text{3}}Characteristic_{i,t-\text{1}}+\gamma {Controls}_{i,t-\text{1}}+{Quarter}_{t}+\lambda_{t}+\epsilon_{i,t}$$ (8)

Where $Characteristic_{i}$ is the dummy variable for fund managers’ characteristics: Degree (1 if the manager holds a Ph.D., 0 otherwise), Gender (1 if female, 0 otherwise), Experience (1 if tenure is less than two years, 0 otherwise), and Fund Size (1 if the fund is in the bottom 30% by size, 0 otherwise).

The regression results in Table 11 reveal significant cross-sectional variations in how fund managers with varying characteristics respond to market crashes in terms of fund risk. Gender and educational background show no statistically significant influence on managers’ risk-taking behavior. Regarding tenure, the interaction term Crash_t-1_ × Tenure_t-1_ is significantly negative at the 5% level, suggesting that fund managers with longer job tenure exhibit a weaker increase in risk-taking after experiencing extreme events. Conversely, managers with shorter tenures act more aggressively post-crisis, likely driven by stronger career concerns and a pressing need to prove their capability. In terms of fund size, the interaction term Crash_t-1_ × TNA_t-1_ is significantly positive at the 5% level, indicating that smaller funds exhibit greater increases in risk following extreme risk events than larger funds. Subject to higher liquidation pressure, smaller funds increase portfolio risk to achieve performance reversals. In contrast, larger funds operate with greater asset diversification and established risk management systems, which constrain their risk-taking behavior [38].

**Table 11:** Manager characteristics.

	(1)	(2)	(3)	(4)
	**Risk**_**t**_			
Crash_t-1_	0.0030^***^	0.0033^***^	0.0034^***^	0.0021^*^
	(2.58)	(2.94)	(2.96)	(1.81)
Crash_t-1_ × Gender_t-1_	−0.0010			
	(−0.45)			
Crash_t-1_ × Degree_t-1_		−0.0036		
		(−1.44)		
Crash_t-1_ × Tenure_t-1_			−0.0023^**^	
			(−1.99)	
Crash_t-1_ × TNA_t-1_				0.0030^**^
				(2.37)
Controls_t-1_	Yes	Yes	Yes	Yes
Individual FE	Yes	Yes	Yes	Yes
Time FE	Yes	Yes	Yes	Yes
_cons	0.1032^***^	0.1032^***^	0.1025^***^	0.1038^***^
	(16.37)	(16.23)	(16.02)	(16.45)
N	61925	61925	62435	61925
R^2^	0.6910	0.6910	0.6906	0.6910

*t* statistics in parentheses.

* *p* < 0.1, ** *p* < 0.05, *** *p* < 0.01.

### 5.6. Future fund performance and flows

In this section, we examine the extended impacts of fund managers’ experience with the 2007–2008 market crash on future fund characteristics, focusing on performance, fees, and flows. We estimate Equation (1) using these outcomes as dependent variables. Performance is captured through five distinct metrics: raw returns (Ret_t_), the Sharpe ratio (SR_t_), the CAPM alpha (Alpha_t_), the Fama-French three-factor alpha (FF3alpha_t_), and the five-factor alpha (FF5alpha_t_). Fund flows (Flow_t_) are measured over the period from t to t + 1.

Table 12 reports the estimation results. Across all performance specifications, the coefficient on Crash_t-1_ is significantly positive. This indicates that crash-exposed managers generate higher raw returns, risk-adjusted returns, and factor alphas in subsequent periods. However, Crash_t-1_ is significantly negative correlated with fund flows (Flow_t_), indicating that despite the higher generated returns, investors allocate less capital to these funds. This suggests that investors discount the outperformance of crash-exposed managers, likely penalizing the concurrent increase in portfolio risk documented earlier.

**Table 12:** Future fund performance and flows.

	(1)	(2)	(3)	(4)	(5)	(6)
	Ret_t_	Alpha_t_	SR_t_	FF3alpha_t_	FF5alpha_t_	Flow_t_
Crash_t-1_	0.0039^**^	0.0016^*^	0.0331^*^	0.0020^***^	0.0018^**^	−0.1730^**^
	(2.40)	(1.78)	(1.84)	(2.65)	(2.23)	(−2.12)
Controls_t-1_	Yes	Yes	Yes	Yes	Yes	Yes
Individual FE	Yes	Yes	Yes	Yes	Yes	Yes
Time FE	Yes	Yes	Yes	Yes	Yes	Yes
_cons	0.0627^***^	0.0007	−0.0352	0.0104	0.0173^**^	2.2537^***^
	(5.51)	(0.08)	(−0.31)	(1.35)	(2.12)	(3.96)
N	61289	50401	61421	50401	50401	61147
R^2^	0.6076	0.7344	0.5145	0.6443	0.5944	0.1635

t statistics in parentheses.

* *p < 0.1,* ** *p < 0.05,* *** *p < 0.01.*

Overall, Crash remains a robust and statistically significant predictor under various controls and fixed effects, lending empirical support to the hypothesis that extreme risk experiences have long-term effects on managerial decision-making.

## 6. Conclusion

Focusing on the Chinese mutual fund market from 2009 to 2024, we employ a two-way fixed-effects panel model to investigate how extreme risk experiences impact managers’ subsequent risk-taking. Contrary to traditional risk aversion assumptions, we find that managers who survive market crashes significantly increase both systematic and idiosyncratic portfolio risks post-crisis. We show this proactive escalation is driven by two mechanisms: portfolio adjustments, specifically heightened industry and equity concentration, and compensation incentives, where surviving a crisis signals capability, intensifies tournament pressures, and increases managerial risk tolerance. We further document that this effect is stronger among small-scale funds, longer-tenured managers, and under highly convex flow-performance relationships. Ultimately, while this aggressive risk-taking enhances returns, the resulting volatility frequently exceeds retail investors’ tolerances, paradoxically triggering short-term capital outflows and highlighting a fundamental principal-agent divergence in risk preferences.

Based on these findings, this study proposes several practical and policy implications. First, regulators and mutual fund companies must reform short-term, “tournament-style” evaluation mechanisms. Performance appraisals should adopt longer assessment windows and prioritize risk-adjusted metrics, such as the Sharpe ratio and maximum drawdown, to mitigate excessive tail-risk taking driven by convex incentives. Second, fund distribution agencies must enhance investor education and product suitability management. Explicitly disclosing volatility and downside risks will foster rational risk-return expectations, helping to alleviate the structural mismatch where improved performance paradoxically triggers capital outflows. Third, amid rising macroeconomic uncertainty, asset management institutions should highly value the risk management capabilities of managers with crisis experience. These managers offer proven advantages in asset pricing and risk identification during extreme market stress. Finally, financial regulatory bodies, such as the Financial Stability Board (FSB), must monitor post-crisis behavioral shifts in institutional risk-taking. Incorporating these behavioral characteristics into macroprudential frameworks and stress testing will better mitigate systemic liquidity risks and cross-market contagion following extreme shocks.
